# A COSMIN systematic review of generic patient-reported outcome measures in Switzerland

**DOI:** 10.1007/s11136-025-03942-x

**Published:** 2025-04-07

**Authors:** Thanh Elsener, Matthew Kerry, Nikola Biller-Andorno

**Affiliations:** 1 Institute of Public Health, ZHAW Zurich University of Applied Sciences, Katharina-Sulzer-Platz 9, Winterthur, 8400 Zurich, Switzerland; 2 Institute of Biomedical Ethics and History of Medicine, University of Zürich, Winterthurerstrasse 30, 8006 Zürich, Switzerland

**Keywords:** Content validity, Measurement properties, Generic PROM, Health-related Quality of life, Switzerland, COSMIN, Systematic reviews

## Abstract

**Purpose:**

To conduct a systematic review of the quality of generic patient-reported outcome measures (PROMs) for clinical or population research or practice using the COnsensus-based Standards for the selection of health Measurement INstruments (COSMIN) methodology, and to derive recommendations for usage of generic PROMs within Switzerland.

**Methods:**

We searched six databases (PubMed, CINAHL, Web of Science, PsycINFO, EMBASE, Cochrane) and grey literature in Switzerland to identify studies reporting on the development or validation of any generic PROMs used in Switzerland. Methodological quality of each study was assessed with COSMIN’s Risk of Bias Checklist. Measurement property evidence (content validity and psychometrics) was further evaluated according to COSMIN’s criteria for good measurement properties. Overall evidence was synthesized according to COSMIN’s modified GRADE approach to generate recommendations for future use or disuse of generic PROMs within Switzerland.

**Results:**

Data from k = 49 studies reporting on five PROMs (EQ-5D, SF-36, PROMIS-29, WHOQOL-BREF, WORQ) were included. Among these, the SF-36 can be recommended for use. The PROMIS-29, WHOQOL-BREF, and WORQ have the potential to be recommended for use, but require further validation. The EQ-5D is not recommendable for future use.

**Conclusion:**

With a limited number of content validity studies, WHOQOL-BREF showed sufficiency with moderate quality, while other PROMs showed mixed quality ranging from very low to moderate. Synthesizing all measurement property evidence, SF-36 was identified as recommendable. PROMIS-29, WHOQOL-BREF, and WORQ were identified as potentially recommendable pending further validation evidence. The EQ-5D was identified as unrecommendable for future use within Switzerland.

**Supplementary Information:**

The online version contains supplementary material available at 10.1007/s11136-025-03942-x.

## Introduction

Patient-reported outcome measures (PROMs), defined as “a report coming directly from patients about how they feel or function without interpretation by healthcare professionals or anyone else” continue to figure prominently in quality-based paradigm shifts of national health systems [1]. In Switzerland, PROMs’ growth is further exhibited by several governmental and professional recommendations toward improving healthcare quality [2–5]. In contrast to Switzerland’s rapidly evolving usage of disease-specific PROMs, however, there is no coherent guidance for usage of generic PROMs [2, 6, 7]. Extrapolating, the literature’s systematic reviews of generic PROMs have largely focused on specific diseases/conditions or patient segments [8], whereas only one study that we are aware of specifically focus geographically for country-specific recommendations [1]. Steinbeck et al. [9] highlighted that, until 2021, projects were initiated from either voluntary bottom-up provider or regional level, mainly focusing on orthopedics and cancer care because of their feasibility. Efforts have been paid to study beyond individual level such as a pilot project from Canton of Wallis, where they collect PRO-data across settings from hospitals, care homes and disease areas. An outstanding example is from the University of Basel, where they have successfully implemented 15 outcome standard sets in areas like orthopedics, cancer care, and chronic diseases. However, sofar, there is no uniform PRO-collection framework and no standard PROM sets that prohibits sustainable implementation with high quality of data. In extreme cases, lack of country-specific generic PROMs has resulted in Switzerland utilizing neighboring or linguistically similar countries’ reference values for comparative effectiveness purposes [10]. Similar to other developed nations’ health systems, given Switzerland’s aging population (20% > age 65) and concomitant multimorbidity-related costs (33%/chronic condition), formulating actionable recommendations for generic PROMs holds great import [11, 12]. Furthermore, the intersection of Switzerland’s advanced, private Bismarckian health-insurance model and its heterogeneous linguistic composition presents a challenge that may be concomitant only to its value-potential for establishing generic-PROM recommendations [13]. Finally, conducting a precedential systematic review within a multi-linguistic country may serve substantive interest to health researchers from other multi-linguistic countries (e.g., Belgium, Canada, India) aiming to render their own PROM recommendations. Fortunately, the COnsensus-based Standards for the selection of health Measurement INstruments (COSMIN) methodology facilitates the process of generating recommendations for PROM usage [14]. Generally, the COSMIN system enables researchers to: (1) Assess methodological quality of PROM development, content validity, and psychometric studies, (2) Evaluate PROMs’s measurement properties (content and psychometric), (3) Grade the overall quality of evidence, and (4) Synthesize all aspects to formulate recommendations. For the current systematic review, therefore, the aim is to identify all previously used generic PROMs that measure health-related quality of life for adults living in Switzerland and evaluate their measurement properties using the COSMIN methodology, summarize evidence, compare and formulate recommendations (recommendable, needing further validation, or unrecommendable) for future usage by clinicians and researchers.

## Methods

### Conductance and reporting protocol

The present systematic review was conducted in accordance to the COSMIN manual Version 1.0 February 2018 [14, 15]. All reporting follows COSMIN guidelines and the recommendations of the Preferred Reporting Items for Systematic Reviews and Meta-Analyses (PRISMA- 2020) statement [16].

**Table 1:** Ten measurement properties assessed in the study

Measurement property	Assessment
*Content validity*	
1. $\text {PROM development}^{a}$ (PROM design, Cognitive interview study or pilot testing):	$\text {Quality}^{*}$
2. Content validity: (Relevance, Comprehensibility, Comprehensiveness)	$$\text {Quality}^{*}$$ $$\text {Rating}^{**}$$
*Internal structure*	
3. Structural validity	$\text {Quality}^{*}$
4. Internal consistency	$$\text {Rating}^{**}$$
5. Cross?cultural validity	
*Remaining*	
7. Measurement error	
8. Criterion validity	
9. Hypotheses testing for construct validity	
10. Responsiveness	

$$\text {PROM development}^{a}$$ is not a measurement property, but taken into account when evaluating content validity. $$^{*}$$: the methodological quality of evidence of single study was rated using COSMIN Risk of Bias with four categories: very good, adequate, doubtful, inadequate, $$^{**}$$: the result of single study for each measurement property was rated against COSMIN good measurement criteria with three categories: sufficient (+), insufficient (−), indeterminate (?)

**Table 2:** Summarized grading the quality of content validity’s evidence

Final grade of quality	Criteria
High	At least one content validity study of very good or adequate
Moderate	At least one content validity study of doubtful quality
	Only content validity studies with inadequate quality of no content validity studies available but with a very good or adequate PROM development study
Low	Only content validity studies with inadequate quality of no content validity studies available but with a doubtful PROM development study
Very low	Only reviewers rating available, no content validity and no PROM development study (or inadequate quality)

**Fig. 1:**
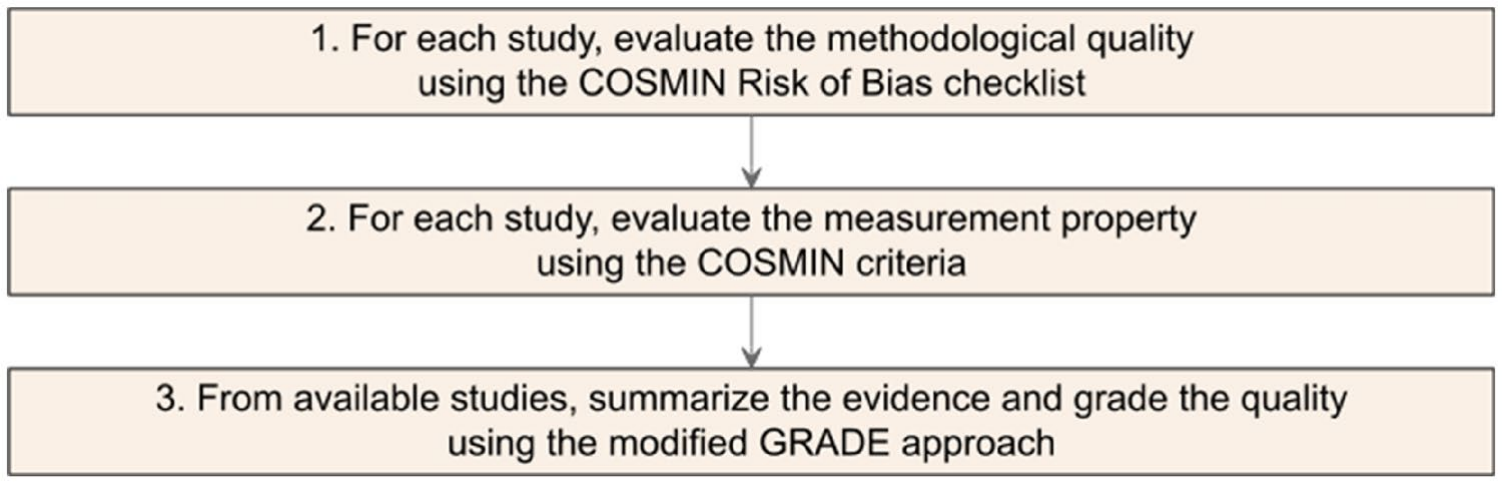
Flow chart summarizing COSMIN evidence synthesis steps

**Fig. 2:**
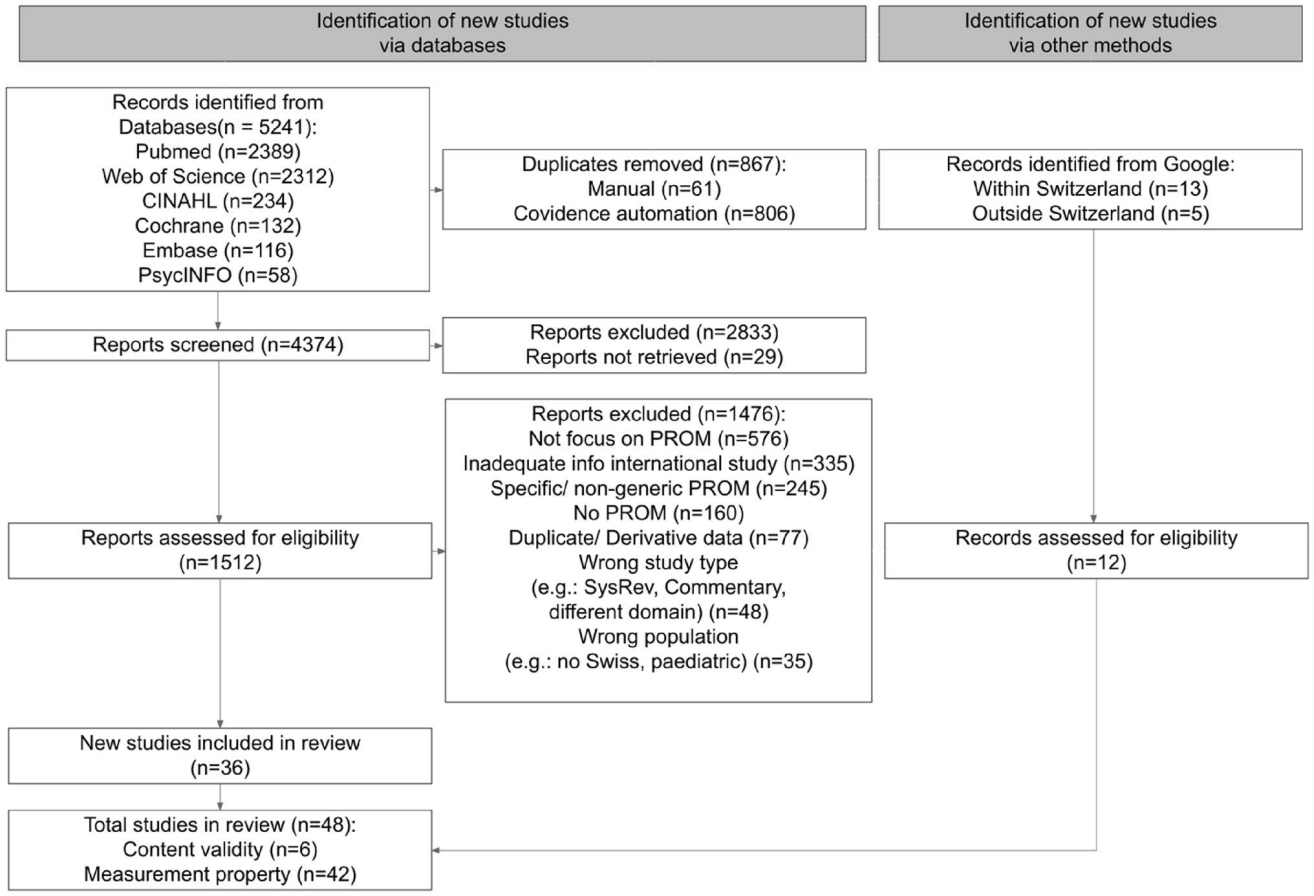
PRISMA flow chart showing the results of study selection process

**Fig. 3:**
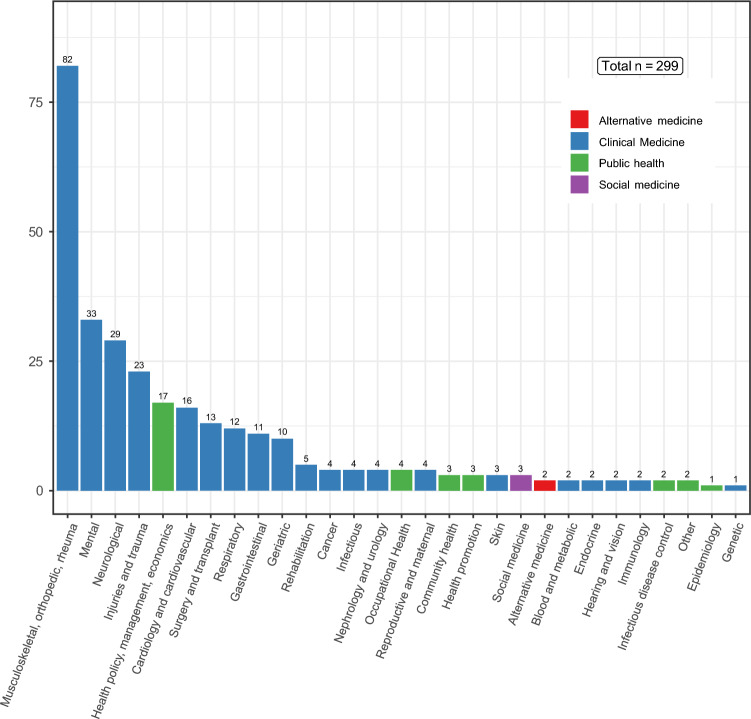
PROM distribution of 299 studies in different sectors of healthcare

### Literature search

Search protocol can be found in the Appendix. In brief, the search strings were composed according to the exclusion filter from Terwee et al. [17] together with Oxford filter and were combined with the keyword ’Switzerland’ or ’Swiss’ and ’patient-reported outcome’. Therefore, this systematic review focused exclusively on studies conducted within Switzerland. Additionally, we also used 17 generic PROMs, identified by Churruca et al. [18] in 2021, and searched from six electronic databases Pubmed, CINAHL, Cochrane, Web of Science, PsycINFO and EMBASE. Children or pediatric population or animal related studies were excluded. There was no restriction to time or language. We aimed to find all possible studies that used generic PROM and were conducted in Switzerland. Primarily, we want to assess the content validity and the measurement properties of generic PROMs used in Switzerland. Secondarily, we want to understand how and where they have been implemented.

### Eligibility criteria

We included only full-text primary research articles published in English, German, French, or Italian. Non-English studies were translated into English using Google Translate. Participants in the included studies were required to be over 18 years of age and residing in Switzerland.

It is important to note that PROMs are one aspect of the superordinate construct, health-related quality of life (HR-QoL) [19]. In our study, only a subset of PROM, the generic HR-QoL measurement, was considered. That is if a generic PROM assessed at least three domains of quality of life: physical, mental, and social. Therefore, we excluded studies that utilized single-item measures of overall quality of life or PROMs assessing fewer domains, as well as specific PROMs focused on only one disease condition.

Eligible studies were required to report on at least one aspect of PROM development, content validity, or a measurement property. Studies that used PROMs solely as secondary instruments or did not provide sufficient information for the Swiss population (e.g., failing to report mean or standard deviation of a PROM score for the Swiss population in international studies) were excluded.

For studies published in multiple journals or using the same cohort with identical PROMs over time, we flagged them as containing duplicated or derivative datasets and included only the studies providing the most comprehensive information (e.g., largest sample size).

### Study selection

First, two reviewers (MK and TE) independently screened titles and articles. After an inter-rater agreement above 80% of screening 200 full-text papers was reached, the reviewers split to screen full-text articles individually. The screening process was tracked by COVIDENCE, a web-based tool for systematic review management. A subset of full-text articles were selected to record the name of used PROM, the health’s domain and sector, and the characteristics of study population.

If information on PROM development or important measurement properties (e.g. Structural validity) were missing, we searched from Google or COSMIN systematic review database for additional studies in neighboring countries such as Germany, France, Italy or EU regions.

### Data extraction and appraisal

For details of information about the COSMIN definitions, methodology, Risk of Bias checklist and content validity checklist, please refer to Prinsen et al. [14], Terwee et al. [15], Mokkink et al. [20]. Data were extracted and stored in Google Sheet using the modified Excel template to document ratings and determine quality ratings provided by the COSMIN website. The PRISMA flowchart was built and descriptive statistics were performed using R (version 4.3.0, http://www.r-project.org) and RStudio (version 2023.03.0).

As shown briefly in Table 1, ten measurement properties were assessed ranging from content validity including PROM development and its three main aspects (relevance, comprehensiveness, comprehensibility) to internal structure and other remaining properties. For each measurement property, the results of individual studies were rated using COSMIN criteria for good content validity and measurement with three categories (sufficient +, insufficient −, indeterminate?). For example, reliability was rated sufficient (+) if one study showed ICC or weighted Kappa ≥ 0*.*70. Additionally, the methodological quality of each study was graded using COSMIN Risk of Bias checklist with four categories (very good, adequate, doubtful, inadequate). Except for PROM development, because it is not a measurement property but is important, we only rated the methodological quality of relevant studies.

Each aspect of PROM development (e.g., PROM design) and content validity (e.g., Relevance) was evaluated separately. In addition to the available evidence from the literature, reviewers also rated the content of the PROM themselves. Reviewer ratings were counted as evidence, but these had lower leverage compared to the content validity study and the PROM development study.

The COSMIN Risk of Bias checklist contains ten boxes evaluating the corresponding methodological quality of each measurement property, with each box containing multiple standards. The overall quality rating of each study on a measurement property was determined applying the ’worst score counts’ principle, which took the lowest rating of any standard in a given box.

The COSMIN User Manual for assessing the content validity of PROMs [15] suggests that in case no content validity studies are available for the target population, researchers may consider including studies conducted in slightly different populations. As a consequence, the quality of evidence should be downgraded to account for this indirectness. Given that Switzerland shares geographical borders and national languages with Germany, France, and Italy, we also reviewed evidence from these neighboring countries. This approach was intended to strengthen the quality of the review, as there was limited research on content validity specifically conducted in Switzerland at the time of conducting this systematic review (2023).

Furthermore, in the assessment of internal structure, COSMIN guideline [14, 15, 17] recommended that structural validity should be evaluated prior to internal consistency, as the interpretation of internal consistency is dependent on the results of structural validity. Evidence supporting structural validity can be gathered from multiple studies, which should be synthesized and evaluated for quality. If limitations such as indirectness are identified, the evidence should be downgraded accordingly. Only after this process is completed should the internal consistency be assessed. Consequently, the quality of evidence for internal consistency cannot exceed that of structural validity. To address gaps in domestic data, researchers seek additional studies from neighboring countries.

### Evidence synthesis

After rating individual studies, the evidence was synthesized for each PROM. The evidence for each measurement property was pooled qualitatively into four categories: sufficient (+), insufficient (−), inconsistent (±) and indeterminate (?). If more than 75% of the individual results were either ‘sufficient’ or ‘insufficient’, the pooled results were considered consistent and qualitatively summarized and rated against the COSMIN criteria once again. In case of inconsistency, the reviewers sought for an explanation. In case of remained unexplained inconsistency, the overall result was rated as ‘inconsistent’ (±). An ‘indeterminate’ (?) rating was given when the individual results were all rated as ‘indeterminate’.

Finally, the quality of the evidence was graded as high, moderate, low or very low evidence using the modified GRADE approach. This approach used four factors to downgrade the quality: (1) risk of bias, (the methodological quality of individual studies), (2) inconsistency (unexplained inconsistency of results across studies), (3) imprecision (small total sample size of the available studies), and (4) indirectness (evidence from different populations than the population of interest). Specifically for content validity, COSMIN provided a simplified process to grade the final quality of evidence after downgrading from the modified GRADE approach, as shown in Table 2. All of the steps mentioned in data extraction & appraisal and evidence synthesis were performed independently by two reviewers (MK and TE). Disagreement was discussed until consensus was reached.

Characteristics of the study population (i.e. age, sex, participant condition characteristics, study setting and design, language and location) of the selected studies for assessing PROM development and content validity or measurement properties were extracted by TE and confirmed by MK.

For a general outline of the COSMIN methodology, Fig. 1 shows four main steps to evaluate a measurement property of a PROM: (1) Risk-of-bias rating, (2) Measurement property assessment, (3) Qualitative pooling across studies, and (4) Evidence quality grading.

### Formulating recommendation

From the final synthesized evidence of all ten measurement properties, COSMIN guideline v.1 recommends to categorize PROMs into three categories: Category (A) includes PROMs having sufficient evidence for content validity with any level of quality and at least low quality evidence for sufficient internal consistency, and hence is recommendable for further use. Category (C) includes PROMs having insufficient evidence of any measurement property with high quality and hence is not recommendable for further use. Category (B) contains PROMs that cannot be classified as (A) or (C) and hence needs further research before further recommendation.

## Results

### Literature search

The search results from multiple databases were imported and stored in COVIDENCE from September 2023 to November 2023. The search resulted in 36 studies in the final selection from 5241 articles. 867 duplicate records were found. 2833 records were excluded after screening title and abstract and 29 could not be retrieved. A total of 1476 full-text articles did not meet the inclusion criteria.

Besides search results from six databases, from Google search, we included finally 13 additional studies that were needed for our data synthesis. These studies provided evidence mainly for content validity and structural validity, since these evidence was not available in Switzerland at the time conducting this review.

Figure 2 presents a PRISMA flow chart o f t he selection process. Of 48 final selected studies, 42 studies were used to extract data for measurement properties and 6 studies for content validity. Evidence for four PROMs (EQ-5D, PROMIS-29, SF-36, WHOQOL-BREF) were found to be in accordance with the inclusion criteria and hence were selected for our further analysis. In addition, we included WORQ, a PROM developed originally for vocational rehabilitation. Even though it only includes functioning questions, it was developed in Switzerland and tested in different groups of patients for various measurement properties.

### Descriptive statistics

First, to answer which generic PROMs have been used and where they have been implemented in Switzerland, descriptive statistics were conducted.

Among the 1476 excluded studies, the majority utilized PROM as a secondary instrument. Specifically, we identified at least 250 studies using various versions of the SF-36, 93 using the EQ-5D, 38 using the WHOQOL-BREF, 30 employing a single-item self-rated health measure, and 29 using the Patient Global Impression of Change. Additionally, 11 studies used locally developed PROMs, which consisted mainly of a few items from a well-described HR-QoL, 8 utilized the Nottingham Health Profile, and a smaller number employed the following questionnaire: WHO-5 (6), PRISM (5), King’s Health Questionnaire (3), Patient Health Questionnaire (3), EUROHIS-QOL (1), McGill Quality of Life Questionnaire (1), Moorehead–Ardelt Quality of Life Questionnaire (2), SEIQoL-DW (1), SEL (1). About one-third of the excluded studies were published after 2019.

Together with the 36 included new selected studies in the finals, we selected a subset of 263 random studies out of 1476 excluded studies to study which health domains generic PROMs have been used in Switzerland. Figure 3 showed their distribution (k = 299) in different health domains. The majority of the studies used generic PROMs in Clinical Medicine, especially in Musculoskeletal, Orthopedic and Rheumatology predictably. However, a wide range of implementation was observed ranging from Mental, Neurological to Epidemiology and Genetic sector. A detailed list of conditions and sectors can be found in the additional extended data. An overall increasing trend of generic PROM usage is shown in the Appendix Fig. B1.

Among 299 studies, 187 explicitly reported the use of language as an inclusion criterion. Among these, 171 studies used national languages (German, French, Italian, or combinations thereof), 8 included English, 3 used Spanish, 2 Albanian, and 1 used Turkish, Arabic, Farsi, or Tamil.

### Content validity

Six studies were analyzed to assess the content validity of five patient-reported outcome measures (PROMs). The characteristics of these six studies and the participants are detailed in Table 3. Two of the studies specifically evaluated the development quality of WHOQOL-BREF and WORQ. For the SF-36, EQ-5D, and PROMIS-29 measures, existing ratings of development quality were sourced from the COSMIN website [21, 22]. The remaining four studies were examined both the rating and the quality of content validity. Detailed ratings, including for development and content validity studies, along with the subjective evaluations of the reviewers, are provided in Table 4. COSMIN rating for quality of evidence and for overall rating was conducted by two sets of boxes separately with different qualitative summarize methods. Therefore, in some cases such as the relevance of SF-36, the quality of a study could be doubtful followed by an undetermined rating.

**Table 3:** Population characteristics for PROM development and content validity studies

PROM	Ref	Year	Age	Settings	Design	Location	Language
PROMIS-29	Kerry et al. [23]	2023	$\mu = 47.35$	Online survey and meeting	4 round digital Delphi Study	Switzerland	German
WORQ	Portmann et al. [24]	2013	$\mu = 29.2$, sd = 11.7	Clinic	Qualitative study with semi-guided interviews	Switzerland	German
	Finger et al. [25]	2013			Cognitive testing	Switzerland	German
WHOQOL-BREF	WHOQOL Group 1998 [26]	1998	< 45 years 50%, 45+ 50%	33 field centers: primary care settings, hospitals and community care settings	Mixed study (qualitative interview, content analysis, and quantitative survey)	International collaborative without Switzerland	30 languages including German, French, Italian
SF-36	Perneger et al. [27]	1992			Pilot testing study	Switzerland	French
EQ-5D, SF-36, WHOQOL-BREF	Rohr et al. [28]	2020	$\mu = 81$	Hospital	Qualitative study with think aloud methodology and semi-standardized interview	Germany	German

**Table 4:** Synthesized evidence for content validity from individual evidence from individual studies and reviewers rating

Ref	Relevance		Comprehensiveness		Comprehensibility	
	Ratings	Quality	Ratings	Quality	Ratings	Quality
WHOQOL-BREF						
Development: [26]	+	V	+	V	+	V
Content validity: [26]	+	V	+	V	+	V
Content validity: [28]	+	A	−	A	+	A
Reviewers	+		+		+	
Overall	+	Moderate	+	Moderate	+	Moderate
SF-36						
Development: [21, 22]		I		I		I
Content validity: [27]	?	D	?	D		
Content validity: [28] $$^{**}$$	+	A	−	A	+	A
Reviewers	+		+		+	
Overall	+	Moderate	+	Low	+	Moderate
$\text {PROMIS-29}^{*}$:						
Development: [21, 22] $^{*}$		D		D		A
Content validity: [23]	?	D	?	D	?	I
Reviewers	+		+		+	
Overall	+	Low	+	Low	+	Moderate
WORQ:						
Development: [25]		I		A		D
Content validity: [24]	?	I	?	I		
Reviewers	+		+		+	
Overall	+	Very low	+	Moderate	+	Low
EQ-5D:						
DV: [21, 22]		I		I		I
Content validity: [28]	+	A	−	A	+	A
Reviewers	+		−		+	
Overall	+	Moderate	−	Moderate	+	Moderate

I: inadequate, D: doubtful, A: adequate, V: very good. $$+$$: Sufficient, −: Insufficient,?: indeterminate, ±: inconsistent. $$^{*}$$: evidence is only available for the versions of PROMIS Physical Function short form. Development: studies collecting evidence for PROM development, Content validity: studies collecting evidence for any of the three aspects of PROM Content validity. $$^{**}$$: [28] used SF-12, a shorter form of SF-36, has only one or two items from each of the eight health concepts of the SF-36

Table 4 presents the synthesized evidence on the content validity of the five PROMs. Overall, because of the limited number of studies available, studies from neighboring countries were search and included to strengthen to quality of our review. However, the grade of quality was downgraded because of its indirectness to the population of interest. The quality of evidence regarding content validity was moderate to very low for most of the assessed PROMs, with the exception of the WHOQOL-BREF. While most ratings were sufficient, the EQ-5D showed insufficient comprehensiveness. This conclusion was drawn from a study conducted on a small sample of elderly German patients with hip fractures [28], a population with physical limitations. Population characteristics of individual studies are detailed in Table 3 in the Appendix. In the case of PROMIS-29, we only found the COSMIN existed PROM development ratings with doubtful to adequate quality for the versions of PROMIS Physical Function short form. As a result, we concluded sufficiency in three aspects with low to moderate quality for the physical domain of PROMIS-29, whereas the remaining domains were assigned very low evidence quality after applying the modified GRADE approach to downgrade the ratings.

### Other measurement properties

In total there were 42 studies assessed the measurement properties of five PROMs. The study characteristics are displayed in Table 5. The results of the ratings for methodological quality and the criteria for evaluating good measurement properties in individual studies are presented in Appendix, Table C2. A synthesis of the evidence for each measurement property of the PROMs, which was qualitatively pooled from individual study results was conducted. These findings were subsequently reassessed against the COSMIN criteria and assigned a grade for the quality of evidence using the modified GRADE approach (Table 6).

**Table 5:** Population characteristics for studies assessing measurement properties

Ref	Age	Settings	Design	Female (%)	n	Location	Language
EQ-5D (k = 12)							
[29]	$\mu =$ 66.8, sd = 8.1	Clinics	RCT	50	123	DE-CH	DE
[30]	group 1: $\mu =$ 67.1, sd = 8.4 group 2: $\mu =$ 69.7, sd = 8.9	Hospital	Longitudinal	45, 60	74, 169	DE-CH	DE, EN, IT, FR
[31]	$\mu =$ 69.1, sd = 10.1	Large teaching hospital	Before–after	62.2	1565	DE-CH	–
[32]	$\mu =$ 64, sd = 10	Orthopedic hospital	Longitudinal	57	454	DE-CH	DE
[33]	$\mu =$ 64, $\left[ 60, 67\right]$	Orthopedic hospital	Prospective cohort, before-after	64	58	DE-CH	DE
[34]	$\mu =$ 80, $\left[ 65, 90+\right]$	Community dwelling	Cross-sectional	48	2888	FR, DE, IT-CH	DE, FR, EN
[35]	$\mu =$ 55.1, sd = 15.2, $\left[ 21, 91\right]$	Rheumatology and manual medicine practices	Before–after	61	96	IT-CH	IT
[36]	$\mu =$ 46.0, sd = 8.4, $\left[ 22.3, 61.9\right]$	Orthopedic hospital	Before–after	54	89	DE-CH	DE
[37]	$\mu =$ 35.9, sd = 11.5	Orthopedic hospital	Before–after	56	102	DE-CH	DE
[38]	$\mu =$ 50.6, sd = 17.4	Regional hospitals	Before–after	6	51		DE
[39]	$\mu =$ 50, $\left[ 18, 80+\right]$	Mail survey	Cross-sectional	56	1952	FR-CH	FR
[40]	$\left[ 16, 93\right]$	Home interview	Cross-sectional	53	2022	DE	DE
PROMIS-29 (k = 6)							
[41]	$\mu =$ 53, sd = 13, $\left[ 21, 96\right]$	Routine clinical practice	Cross-sectional	59	63602	NL	NL
[42]	$\mu =$ 68.5, sd = 9	Hospital	Before–after	59	119	DE-CH	DE
[43]	$\mu =$ 55.4, sd = 14.2	Hospital	Before–after	69	202	DE-CH	DE
[44]	$\mu =$ 65.9, sd = 10.2	Hospital	Before–after	51	116	DE-CH	DE
[45]	$\left[ 18,89\right]$, med = 56	Online survey	Cross-sectional	52	14098	US	EN
[46]	$\mu =$ 51.2, sd = 12.9	Outpatient clinic	Cross-sectional	90	71	DE-CH	DE
SF-36 (k = 16)							
[47]	$\mu =$ 43, sd = 14	Online survey	Cross-sectional	76	1581	FR, DE, IT-CH	DE, FR, IT
[48]	$\mu =$ 48, sd = 12.7	Rehabilitation clinic	Prospective cohort, before-after	59	177	DE-CH	DE
[49]	$\mu =$ 49, $\left[ 18, 95\right]$	Questionnaire envelope	Cross-sectional	58	1209	FR, DE, IT-CH	DE, FR, IT
[50]	$\mu =$ 66.1, sd = 10.2	Rehabilitation clinic	Prospective cohort, before-after	78	190	DE-CH	DE
[51]	$\mu =$ 19.95, sd = 1.19	Military recruitment centers	Cross-sectional	0	48	DE, FR-CH	DE, FR
[52]	$\mu =$ 61.9, sd = 13, $\left[ 34.4, 87.2\right]$	Orthopedic hospital	Before–after	71	65	DE-CH	DE
[53]	$\mu =$ 67, sd = 9	Orthopedic hospital	Before–after	61	79	DE-CH	DE
[54]	$\mu =$ 40.67, sd = 9.66	Local police force	Cross-sectional	25	460	DE-CH	DE
[55]	$\mu =$ 67.3, sd = 10.4, $\left[ 39.5,88.5\right]$	Orthopedic hospital	Before–after	65	138	DE-CH	DE
[56]	$\mu =$ 46.3, sd = 10.5	Rehabilitation clinic	Before–after	80	273	DE-CH	DE
[57]	$\mu =$ 47.2, sd = 14.2, $\left[ 18, 60\right]$	Interview at local centers	Cross-sectional	53	216	FR, DE-CH	–
[58]	$\mu =$ 68.3, sd = 9.9, $\left[ 40, 90\right]$	Inpatient rehabilitation	Before–after	48	114	DE-CH	DE
[59]	$\mu =$ $\left[ 41,47\right]$	Mixed settings: survey	Cross-sectional	50	$\text {n}_\text {FR} = 3,656$, $\text {n}_\text {DE} = 2,914$, $\text {n}_\text {IT} = 2,031$	DK, FR, DE, IT, NL, NO, ES, SE, GB, US	–
[60]	Men: $\mu =$ 48,3, sd = 9.9, $\left[ 26, 78\right]$, women: $\mu =$ 46.5, sd = 8.1, $\left[ 29, 66\right]$	Mixed settings: in-patient in hospital, outpatient in care center, social service	Prospective cohort	23	147	FR-CH	FR
[61]	$\mu =$ 30, $\left[ 18, 44\right]$	Mail survey	Prospective cohort		851	FR-CH	FR
[27]	$\mu =$ 30, $\left[ 18, 44\right]$	Mail survey	Cross-sectional	53	1007	FR-CH	NA
WHOQOL-BREF (k = 6)							
[62]	$\mu =$ 55.5, sd = 12, $\left[ 26, 83\right]$	Home	RCT	60	108	SwiSCI	DE
[63]	$\mu =$ 79.6, sd = 6.3, $\left[ 63, 93\right]$	University geriatric outpatient-center	RCT	52	54		DE
[64]	$\mu =$ 36.3, $\left[ 18, 65\right]$	Online therapy	Longitudinal interventional clinical trial	77	252		–
[65]	$\mu =$ 40, $\left[ 15, 60\right]$	National household survey with telephone interview	Cross-sectional	52	10038	FR, DE, IT-CH	DE, FR, IT
[35]	$\mu =$ 55.1, sd = 15.2, $\left[ 21, 91\right]$	Rheumatology and manual medicine practices	Before-after	61	96	IT-CH	IT
[66]	$\mu =$ 45, sd = 16, $\left[ 12, 97\right]$	Mixed settings including healthy and sick people	Cross-sectional		11830	23 countries without Switzerland	
WORQ (k = 3)							
[67]	$\mu =$39.96, sd = 12.9	Outpatient physical therapy clinic	Cross-sectional	61	51	DE-CH	DE
[68]	$\mu =$43.47, sd = 10.9	Rehabilitation teaching hospital	Longitudinal	11	221	FR-CH	FR
[69]	$\mu =$44, $\left[ 41.7, 46.4\right]$	Rehabilitation teaching hospital	Cross-sectional	8	89	FR-CH	FR

Studies are organized in descending order of publication year. $\mu$: mean, sd: standard deviation, $\left[ min, max\right]$: range of values. BA: before-after study, RCT: randomized control trial; DE-, FR-, IT-CH: German, French, Italian-speaking areas of Switzerland; DK: Denmark, FR: France, DE: Germany, IT: Italy, NL: Netherlands, NO: Norwegian, ES: Spain, SE: Sweden, GB: Great Britain, US: USA, CH: Switzerland

**Table 6:** Synthesized evidence for other measurement properties

PROM	Measurement Property	Synthesized result	Overall rating	Quality of evidence
SF-36	Structural validity	CFI > 0.95	+	Moderate
(k = 16)	Internal consistency	Cronbach’s $\alpha = 0.85, \in \left[ 0.7, 0.93 \right]$	+	Moderate$^{*}$
	Reliability	$\text {ICC}_{mean} = 0.74, \in \left[ 0.28, 0.9 \right]$	+	High
	Measurement error	$\text {SEM}_{PCS} = 3.5$, $\text {SEM}_{MCS} = 2.9,$		
		$\text {MDC}_{PCS} = 9.7$, $\text {MDC}_{MCS} = 8.0$	?	
	Construct validity	87.5% studies (7/8) confirmed	+	High
	Responsiveness	89% studies (8/9) confirmed	+	High
EQ-5D	Structural validity	RMSEA = 0.06, GFI = 0.976	+	Moderate
(k = 12)	Internal consistency	Cronbach’s $\alpha$ 0.83 (one sided CI: 0.77)	+	Very low
	Reliability	$\text {ICC}_{mean} = 0.8,\in \left[ 0.67, 0.88 \right]$	+	Low
	Measurement error	Reported MIC $\in \left[ 0.027, 0.209 \right]$	−	High
	Construct validity	86% studies (6/7) confirmed	+	High
	Responsiveness	87.5% studies (7/8) confirmed	+	High
WHOQOL-BREF	Structural validity	RMSEA = 0.07, CFI $\in \left[ 0.86, 0.87 \right]$ for 4-domain model	−	Moderate
(k = 6)	Internal consistency	Cronbach’s $\alpha$ $\in \left[ 0.48, 0.83 \right]$	?$^{*}$	
	Reliability	$\text {ICC}_{mean} = 0.87, ~\in ~\left[ 0.79, 0.92 \right]$	+	Very low
	Measurement error	SEM (Whole) = 0.72	?	Very low
	Construct validity	100% (3/3) confirmed	+	High
	Responsiveness	67% studies (2/3 confirmed negative	?	Moderate
PROMIS (k = 6)	Structural validity	$\text {CFI}~\in ~\left[ 0.95, 0.994 \right]$, $\text {RMSEA}~\in ~\left[ 0.04, 0.1 \right]$	+	High
	Internal consistency	Cronbach’s $\alpha$ 0.89	+	High
	Reliability	$\text {ICC}_{mean} = 0.85,\in \left[ 0.69, 97 \right]$	+	High
	Measurement error	ROC-based: $\text {MIC}_{PAIN}~=~4$, $\text {MIC}_{PI}~=~5$, $\text {MIC}_{PF}~=~4.6$		
		Anchor-based: $\text {MIC}_{PAIN}~=~10$, $\text {MIC}_{PI}~=~8.56$, $\text {MIC}_{PF}~=~7.79$	?	
	Construct validity	100% (3/3) confirmed	+	High
WORQ (k = 3)	Structural validity	No clear violation in unidimensionality, local independence, monotonicity and adequate model fit	+	High
	Internal consistency	Cronbach’s $\alpha$ $\in \left[ 0.84, 0.968\right]$, $\text {PSI}> 0.86$	$+$	High
	Reliability	ICC = 0.935, CI (0.889–0.963)	+	Moderate
	Construct validity	100% (2/2) confirmed	+	High

CFI: Comparative Fit Index, RMSEA: Root Mean Square Error of Approximation, GFI: Goodness of Fit, PSI: Person Separation Index, ICC: Intraclass Correlation coefficient, SEM: Standard Error of Measurement, SDC: Smallest Detectable Change, MDC: Minimal Detectable Change, ROC: Receiver Operator Curve. $\mu$: mean, sd: standard deviation, $\left[ min, max\right]$: range of values. PCS: Physical component score, MCS: Mental component score, PAIN: pain intensity, PI: pain interference, PF: physical function

*Quality of evidence was downgraded due to quality of evidence of internal consistency can not be higher than structural validity. Detail ratings can be found in the appendix

### Formulating recommendation

SF-36 has shown sufficient content validity (with moderate quality for relevance and comprehensibility and low quality for comprehensiveness) and sufficient internal consistency with moderate quality of evidence. Therefore, SF-36 is classified as category A and is recommendable for further use.

On the other hand, EQ-5D has shown insufficient content validity with moderate quality as well as insufficient measurement error with high quality, falls in the category (C) according to COSMIN guideline v1.0 and hence it is not recommendable for further use.

WHOQOL-BREF has sufficient content validity with overall moderate quality of evidence. However, there was insufficient structural validity with a moderate quality of evidence, the internal consistency has shown a wide range of Chronbach’s *α*, and the lower number of available studies, it is concluded that WHOQOL-BREF remains in category B and requires further studies for any further recommendation.

PROMIS-29 was classified a s B. Because the physical function domain demonstrated sufficient content validity with lo w to moderate quality evidence, along with high-quality evidence for sufficiency in other measurement properties. However, other domains suffer from the lack of evidence. In this systematic review, only four studies from Switzerland have been published between 2019 and 2023.

Lastly, WORQ, with a very limited number of found evidence (k = 3), was categorized B because of its sufficiency in content validity and internal structure. It requires more longitudinal studies for responsiveness validation. However, as it only contains functioning questions, it cannot be used to measure health-related quality of life, rather than a tool for measuring work-related quality of life.

## Discussion

This COSMIN systematic review provides precedential recommendations for generic-PROMs usage in Switzerland. The generic PROM in this systematic reviews concerned only HR-QOL tool, that measure at least three aspects: physical, mental and social health.

In our study, we found that different health sectors have been utilizing and benefiting from generic PROMs primarily from Clinical Medicine extending to public health, social medicine and alternative medicine. Our research has also shown that Switzerland has a tendency of increasingly utilizing generic PROM. Many generic PROMs have been implemented but through years, there is a convergence towards SF-36, EQ-5D, WHOQOL-BREF. SF-36, EQ-5D-3L and WHOQOL-BREF were first developed and published between 1990 and 2000 where PROMIS initiative was first developed in 2004 with PROMIS-29 first published in 2 010 [26, 70–72]. The observed time shift in our study could be a result of the know-how transfer, implementation process, translation and cross-culture and other psychometrics validation.

Although diverse stakeholder interest for a generic PROM in Switzerland has been explored, empirical evidence-based recommendations were absent [23]. Although SF-36, EQ-5D have been long introduced and validated in Switzerland, some back before 2010 [39, 47, 49, 73], no up-to-date systematic review has been conducted according to the present-day standard to standardize generic PROM usage in Switzerland, to suggest recommendations for the national generic PROM program. The current study identified five generic instruments previously used in Switzerland. Our findings are elaborated below according to the following COSMIN evaluative criteria for focusing future research efforts: (1) Content validity, (2) Internal structure, and (3) Additional measurement properties [20].

First, regarding development and content validity, it is notable that the vast majority of generic PROMs exhibited low to moderate quality of content validity that rely on studies from other countries [21, 22, 28]. An exception is the WHOQOL-BREF, which exhibited moderate content validity [26], due to the global cross-cultural adaption effort with large focus on qualitative method in 1998. Encouragingly, qualitative research has begun systematically examining patient-reported experiences, and these data may be utilized to yield insights into further generic PROM development and content validation efforts [74]. EQ-5D has showed in Rohr et al. [28] to have insufficiency in content validity in a group of German geriatric patients with physical limitation. In Switzerland, where population is aging rapidly with more than 60% are German speakers, for population wise implementation, it is recommended that EQ-5D should be adapted and tested again before use.

Second, regarding internal structure, SF-36 and WORQ exhibited the highest evidence, whereas WHOQOL-BREF exhibited the lowest evidence. Auspicious for future research in Switzerland is the measurement property of cross-cultural validity. Of all our studies, only two examined measurement invariance and the evidence for structural validity for all PROMs except for WORQ was borrowed from studies outside of Switzerland. This will be critical to future linguistic-/cultural-validation efforts, and should be guided by modern psychometrics, such as item response theory. We are currently undertaking such research in a Swiss-nationally representative cross-sectional survey. Interestingly, although the use of official-language translations were prominent, there was a disproportionate absence of English-based generic PROMs. Switzerland has about 27% non-Swiss residents and English is the most widely spoken non-official language in Switzerland, with 45% of the population using it regularly [75]. This absence of English-based generic PROMs could inhibit the collection and interpretation of QOL of a large subgroup of Swiss population and could not reflect their perspective. Hence, this could be critical for researching migrant, urban, or high-system usage populations [11].

Third, regarding additional measurement properties, evidence varied considerably. For example, across all PROMS, construct validity as reported most often, whereas reliability and especially measurement error were reported least often as shown in Table 7. Furthermore, SF-36 exhibited highest quality evidence, whereas WHOQOL-BREF exhibited lowest quality of evidence. Stephan et al. [42–44] proved PROMIS short forms for pain and function to have sufficiency with high quality. This provides partial confidence for further validation of the full version of PROMIS-29. In constructive critique, we observed partial conflation with properties of reliability, measurement error, and responsiveness, and hope that these properties may be better delineated in impending updates to the COSMIN criteria.

**Table 7:** Occurrence frequency of reported additional measurement properties in 35 selected studies

Measurement property	Studies reporting quality	Studies reporting ratings
Construct validity	25	24
Responsiveness	23	22
Reliability	16	15
Measurement error	6	6
Total studies	35	35

### Limitations

This study has several limitations. First, the true value of PROM usage may be under-estimated due to the relatively low number of included studies and 29 studies could not be retrieved, which might not fully reflect the broader literature. Notably, 10 of these 29 studies were published prior to 2000, and 23 were published before 2017. Based on their titles and abstracts, it is likely that these studies primarily utilized PROMs as measurement tools rather than focusing on reporting their measurement properties and hence likely fall into the exclusion criteria.

Second, our search strategy may have missed relevant studies, especially those not indexed in the selected databases or using different terminology. Finally, we may have overlooked unpublished or grey literature, such as dissertations and conference proceedings. It is also important to note that linguistic translation is distinct from cultural adaptation, and we encourage future iterations of the COSMIN guidelines to highlight this in future interactions [76]. This could introduce publication bias and limit the generalizability of our findings.

Third, this systematic review was conducted and submitted before PRISMA-COSMIN was published in August 2024 Elsman et al. [77]. As a result, the methodology and reporting standards employed in this review may not fully align with the latest best practices outlined in PRISMA-COSMIN. Future updates or revisions of this work would benefit from adhering to these newly established guidelines to ensure methodological rigor and consistency with current norms. Finally, we must note that, terminologically, some researchers accord that PROMs are restricted to patient respondents, whereas public health usage in general populations are more aptly labeled profiles or generic HRQoL measures. This distinction is not critical to the purview of the current systematic review, but the principle may be of importance to some researchers.

## Conclusion

Following the COSMIN guidelines v1.0 together with up-to-date evidence collected from Roser et al. [47, 49], the SF-36 is recommendable for further use for population scale measurement for HR-QoL.

Given the high-quality evidence of insufficient measurement error and moderate-quality evidence of inadequate comprehensiveness, the EQ-5D is recommended to be revised and adapted and currently is not recommended for continued use for population scale measurement.

The WHOQOL-BREF requires further validation of its internal consistency before it can be recommended. While the PROMIS-29 shows promising results for a subset of items, supported by its modern IRT-based model, additional evidence is needed to validate the full 29-item version. WORQ is reviewed here in this study because it was developed in Switzerland, shows to have potential and requires further validation. However, it is worth to point out again that it is a work-related QoL.

As with all validation evidence, this review should be updated to reflect the growing empirical information made available.

## Supplementary Information

Below is the link to the electronic supplementary material.

Supplementary file1 (DOCX 82 kb)

## Data Availability

Interested readers can contact authors for more detailed data.

## Abbreviations

PROM: Patient-reported outcome measure
HR-QoL: Health related quality of life
COSMIN: COnsensus-based Standards for the selection of health Measurement Instruments
SF-36: 36-Item short form health survey
EQ-5D: 5-Item European Quality of Life 5 Dimensions
WHOQOL-BREF: 26-Item World Health Organization Quality of Life Brief Version
PROMIS: 29-Item patient-reported outcomes measurement information system
WORQ: 42-Item work rehabilitation questionnaire
WHO-5: The World Health Organisation-five well-being index
PRISM: Pictorial representation of illness and self measure
EUROHIS-QOL: 8-Item European Health Interview Survey-Quality of Life
SEIQoL-DW: Schedule for the evaluation of individual quality of life
SEL: Skalen zur Erfassung der Lebensqualit¨at

## References

[1] Voshaar, M.O., Terwee, C.B., Haverman, L., Kolk, B., Harkes, M., Woerden, C.S., Breda, F., Breukink, S., Hoop, I., Vermeulen, H., Graaf, E., Hazelzet, J., Leiden, B., Stienen, J., Hoekstra, M., Bart, H., Bommel, H., Determann, D., Verburg, M., Wees, P., Beurskens, A.J. (2023). Development of a standard set of pros and generic proms for dutch medical specialist care: ecommendations from the outcome-based healthcare program working group generic proms.* Quality of Life Research, 32*. 10.1007/s11136-022-03328-3 10.1007/s11136-022-03328-3 PMC10172289 36757571

[2] Vogel, J., Sapin, M., Kuklinski, D., Walker, C., Mantwill, S., Havranek, M., Sabariego, C., Pietro, C.D., Burgstaller, J., Peytremann-Bridevaux, I., & Geissler, A. (2024). Quality monitoring and public reporting: recommendations for the Swiss Healthcare System. Report Mandated by the Federal Quality Commission. Federal Quality Commission. Bern. https://www.bag.admin.ch/dam/bag/de/dokumente/kuv-leistungen/eqk/Quality%20Monitoring%20and%20Public.pdf.download.pdf/Quality%20Monitoring%20and%20Public%20Reporting% 20Recommendations%20for%20the%20Swiss%20Healthcare%20System.pdf

[3] Vincent, C., & Staines, A.: Enhancing the quality and safety of Swiss Healthcare. A National Report Commissioned by the FOPH on the Quality and Safety of Healthcare in Switzerland. 10.13140/RG.2.2.22966.04160

[4] Christoph, A. M. (2019). Variations of care and diagnosis as markers for the quality of medical care in Switzerland.* The Swiss Medical Weekly*. 10.4414/smw.2019.20029 10.4414/smw.2019.20029 30769344

[5] Allegranzi, B., Bjorn, B., Burnand,B., Chopard, P., Conen, D., Pfaff, H., Spirig, R., Staines, A., Vincent, C., & Windeler, J. (2017). Qualit¨at und Sicherheit der Schweizerischen Gesundheitsversorgung Verbessern. Empfehlungen und Vorschl¨ageFu¨r die Bundesstrategie; 2. Bericht. https://www.bag.admin.ch/dam/bag/de/dokumente/kuv-leistungen/qualitaetssicherung/second-report-advisory-board-30-06-2017.pdf.download.pdf/second-report-advisory-board-30-06-2017-de.pdf

[6] Henchoz, Y., Büla, C., Guessous, I., Goy, R., Dupuis, M., & Santos-Eggimann, B. (2020). Validity of the older people quality of life-7 domains (oqol-7) scale.* Health and Quality of Life Outcomes, 18*. 10.1186/s12955-020-01589-5 10.1186/s12955-020-01589-5 PMC7557060 33054841

[7] Saxer, F., Degen, M., & Brodbeck, D. (2020) Implementing systematic outcome assessments in a Swiss healthcare practice: points to consider (2020). https://smw.ch/index.php/smw/announcement/view/36

[8] Piontek, K., Gabes, M., Kann, G., Fechtner, M., & Apfelbacher, C. (2024). Quality of patient-reported outcome measures for primary dysmenorrhea: a systematic review.* Quality of Life Research, 33*. 10.1007/s11136-023-03517-8 10.1007/s11136-023-03517-8 PMC10784326 37902914

[9] Steinbeck, V., Ernse, S., & Pross, C. (2022) Patient-reported outcome measures—an international comparison challenges and success strategies for the implementation in Germany. 10.11586/2021048.

[10] Tasiemski, T., Kujawa, J., Tederko, P., Rubinelli, S., Middleton, J.W., Craig, A., & Post, M. W. M. (2023). Relationship between secondary health conditions and life satisfaction in persons with spinal cord injury: study across twenty-one countries.* Quality of Life Research, 32*. 10.1007/s11136-023-03376-3 10.1007/s11136-023-03376-3 PMC10241701 36862301

[11] Bähler, C., Huber, C.A., Bru¨ngger, B., & Reich, O. (2015). Multimorbidity, health care utilization and costs in an elderly community-dwelling population: A claims data based observational study.* BMC Health Services Research, 15*. 10.1186/s12913-015-0698-2 10.1186/s12913-015-0698-2 PMC4307623 25609174

[12] Colombier, C. (2018). Population ageing in healthcare–a minor issue? Evidence from Switzerland.* Applied Economics, 50*. 10.1080/00036846.2017.1374538

[13] Wallace, L. S. (2013). A view of health care around the world.* Annals of Family Medicine*. 10.1370/afm.1484 23319511 10.1370/afm.1484 PMC3596027

[14] Prinsen, C. A. C., Mokkink, L. B., Bouter, L. M., Alonso, J., Patrick, D. L., Vet, H. C. W., & Terwee, C. B. (2018). Cosmin guideline for systematic reviews of patient-reported outcome measures.* Quality of Life Research, 27*10.1007/s11136-018-1798-3 10.1007/s11136-018-1798-3 PMC5891568 29435801

[15] Terwee, C. B., Prinsen, C. A. C., Chiarotto, A., Westerman, M. J., Patrick, D. L., Alonso, J., Bouter, L. M., Vet, H. C. W., & Mokkink, L. B. (2018). COSMIN methodology for evaluating the content validity of patient-reported outcome measures: A Delphi study.* Quality of Life Research*. 10.1007/s11136-018-1829-0 29550964 10.1007/s11136-018-1829-0 PMC5891557

[16] The PRISMA 2020 statement: An updated guideline for reporting systematic reviews (2021). 10.1136/bmj.n71 10.1136/bmj.n71 PMC8005924 33782057

[17] Terwee, C. B., Jansma, E. P., Riphagen, I. I., & Vet, H. C. W. D. (2009). Development of a methodological pubmed search filter for finding studies on measurement properties of measurement instruments.* Quality of Life Research, 18*. 10.1007/s11136-009-9528-5 10.1007/s11136-009-9528-5 PMC2744791 19711195

[18] Churruca, K., Pomare, C., Ellis, L. A., Long, J. C., Henderson, S. B., Murphy, L. E. D., Leahy, C. J., & Braithwaite, J. (2021). Patient-reported outcome measures (PROMs): A review of generic and condition-specific measures and a discussion of trends and issues.* Health Expectations*. 10.1111/hex.13254 10.1111/hex.13254 PMC8369118 33949755

[19] Bull, C., Teede, H., Watson, D., & Callander, E. J. (2022). Selecting and Implementing Patient-Reported Outcome and Experience Measures to Assess Health System Performance. *JAMA Health Forum*. 10.1001/jamahealthforum.2022.0326 36218960 10.1001/jamahealthforum.2022.0326

[20] Mokkink, L. B., Vet, H. C. W., Prinsen, C. A. C., Patrick, D. L., Alonso, J., Bouter, L. M., & Terwee, C. B. (2018). Cosmin risk of bias checklist for systematic reviews of patient-reported outcome measures.* Quality of Life Research, 27*. 10.1007/s11136-017-1765-4 10.1007/s11136-017-1765-4 PMC5891552 29260445

[21] Chiarotto, A., Terwee, C. B., Kamper, S. J., Boers, M., & Ostelo, R. W. (2018). Evidence on the measurement properties of health-related quality of life instruments is largely missing in patients with low back pain: A systematic review.* The Journal of Clinical Epidemiology*. 10.1016/j.jclinepi.2018.05.006 29793009 10.1016/j.jclinepi.2018.05.006

[22] Chiarotto, A., Ostelo, R. W., Boers, M., & Terwee, C. B. (2018). A systematic review highlights the need to investigate the content validity of patient-reported out-come measures for physical functioning in patients with low back pain.* Journal of Clinical Epidemiology*. 10.1016/j.jclinepi.2017.11.005 29154811 10.1016/j.jclinepi.2017.11.005

[23] Kerry, M. J., Volken, T., Biller-Andorno, N., Gl¨assel, A., & Melloh, M. (2023). A swiss digital delphi study on patient-reported outcomes.* Swiss Medical Weekly, 153* (2023). 10.57187/smw.2023.40125 10.57187/smw.2023.40125 37988458

[24] Portmann, R., Escorpizo, R., Staubli, S., & Finger, M. E. (2014). Content validity of the work rehabilitation questionnaire-self-report version worq-self in a subgroup of spinal cord injury patients.* Spinal Cord, 52*. 10.1038/sc.2013.129 10.1038/sc.2013.129 24247564

[25] Finger, M. E., Escorpizo, R., Bostan, C., & Bie, R. D. (2014). Work rehabilitation questionnaire (worq): Development and preliminary psychometric evidence of an ICF-based questionnaire for vocational rehabilitation.* Journal of Occupational Rehabilitation, 24*. 10.1007/s10926-013-9485-2 10.1007/s10926-013-9485-2 24281830

[26] WHOQOL Group 1998: Programme on mental health: WHOQOL user manual. Technical documents (1998)

[27] Perneger, T. V., Lepl`ege, A., Etter, J. F., & Rougemont, A. (1995). Validation of a French-language version of the mos 36-item short form health survey (sf-36) in young healthy adults.* Journal of Clinical Epidemiology, 48*. 10.1016/0895-4356(94)00227-H 10.1016/0895-4356(94)00227-h 7775992

[28] Rohr, M., Brandstetter, S., Plomer, A.S., Loss, J., Kretschmer, R., Apfelbacher, C.: A qualitative study exploring content validity and feasibility of frequently used generic health-related quality of life measures in older people with hip fracture: The patients’ perspective. Injury **52** (2021). 10.1016/j.injury.2020.09.061 10.1016/j.injury.2020.09.061 33039180

[29] Frei, A., Radtke, T., Lana, K.D., Brun, P., Sigrist, T., Spielmanns, M., Beyer, S., Riegler, T. F., Büsching, G., Spielmanns, S., Kunz, R., Cerini, T., Braun, J., Tomonaga, Y., Serra-Burriel, M., Polhemus, A., Puhan, M. A. (2022). Effectiveness of a long-term home-based exercise training program in patients with copd after pulmonary rehabilitation: A multicenter randomized controlled trial.* Chest, 162*. 10.1016/j.chest.2022.07.026 10.1016/j.chest.2022.07.026 35952766

[30] Wendelspiess, S., Kaelin, R., Vogel, N., Rychen, T., & Arnold, M. P. (2022). No difference in patient-reported satisfaction after 12 months between customised individually made and off-the-shelf total knee arthroplasty.* Knee Surgery, Sports Traumatology, Arthroscopy, 30*. 10.1007/s00167-022-06900-z 10.1007/s00167-022-06900-z PMC9418302 35149877

[31] Giesinger, K., Giesinger, J., Hamilton, D., Rechsteiner, J., & Ladurner, A. (2021). Higher body mass index is associated with larger postoperative improvement in patient-reported outcomes following total knee arthroplasty.* BMC Musculoskeletal Disorders, 22*. 10.1186/s12891-021-04512-1 10.1186/s12891-021-04512-1 PMC8310599 34303341

[32] Marks, M., Grobet, C., Audige, L. (2021). Validity, responsiveness and minimal important change of the eq-5d-5l in patients after rotator cuff repair, shoulder arthroplasty or thumb carpometacarpal arthroplasty.* Quality of Life Research, 30*. 10.1007/s11136-021-02849-7 10.1007/s11136-021-02849-7 PMC8481200 33973108

[33] Marti, C., Hensler, S., Herren, D. B., Niedermann, K., & Marks, M. (2016). Measurement properties of the euroqol eq-5d-5l to assess quality of life in patients undergoing carpal tunnel release.* Journal of Hand Surgery: European Volume, 41*. 10.1177/1753193416659404 10.1177/1753193416659404 27435748

[34] Luthy, C., Cedraschi, C., Allaz, A. F., Herrmann, F. R., Ludwig, C. (2015). Health status and quality of life: results from a national survey in a community-dwelling sample of elderly people.* Quality of Life Research, 24*. 10.1007/s11136-014-0894-2 10.1007/s11136-014-0894-2 25510216

[35] Mannion, A.F., Boneschi, M., Teli, M., Luca, A., Zaina, F., Negrini, S., Schulz, P. J. (2012). Reliability and validity of the cross-culturally adapted italian version of the core outcome measures index.* European Spine Journal, 21*. 10.1007/s00586-011-1741-6 10.1007/s00586-011-1741-6 PMC3535210 21409562

[36] Fankhauser, C. D., Mutter, U., Aghayev, E., & Mannion, A. F. (2012). Validity and responsiveness of the core outcome measures index (comi) for the neck.* European Spine Journal, 21*. 10.1007/s00586-011-1921-4 10.1007/s00586-011-1921-4 PMC3252436 21858567

[37] Impellizzeri, F. M., Mannion, A. F., Naal, F. D., Hersche, O., & Leunig, M. (2012). The early outcome of surgical treatment for femoroacetabular impingement: Success depends on how you measure it.* Osteoarthritis and Cartilage, 20*. 10.1016/j.joca.2012.03.019 10.1016/j.joca.2012.03.019 22469846

[38] Staerkle, R. F., & Villiger, P. (2011). Simple questionnaire for assessing core outcomes in inguinal hernia repair.* British Journal of Surgery, 98*. 10.1002/bjs.7236 10.1002/bjs.7236 20814965

[39] Perneger, T. V., Combescure, C., Courvoisier, D. S. (2010). General population reference values for the French version of the Euroqol eq-5d health utility instrument.* Value in Health, 13*. 10.1111/j.1524-4733.2010.00727.x 10.1111/j.1524-4733.2010.00727.x 20412541

[40] Clouth, J., Brähler, E., Schmidt, P., & Kohlmann, T. (2008). Testing construct validity of EQ-5D by confirmatory factor analysis and structural equation modeling (SEM). In: *Addendum to EuroQol 2007 proceedings*. https://eq-5dpublications.euroqol.org/download?id=0_53699&fileId=54114

[41] Elsman, E. B. M., Roorda, L. D., Smidt, N., Vet, H. C. W., & Terwee, C. B. (2022). Measurement properties of the Dutch promis-29 v2.1 profile in people with and without chronic conditions.* Quality of Life Research, 31*. 10.1007/s11136-022-03171-6 10.1007/s11136-022-03171-6 PMC9587921 35751760

[42] Stephan, A., Stadelmann, V. A., Preiss, S., Impellizzeri, F. M. (2023). Measurement properties of promis short forms for pain and function in patients receiving knee arthroplasty.* Journal of Patient-Reported Outcomes, 7*. 10.1186/s41687-023-00559-x 10.1186/s41687-023-00559-x PMC9975126 36854937

[43] Stephan, A., Mainzer, J., Kümmel, D., & Impellizzeri, F. M. (2019). Measurement properties of promise short forms for pain and function in orthopedic foot and ankle surgery patients.* Quality of Life Research, 28*. 10.1007/s11136-019-02221-w 10.1007/s11136-019-02221-w 31177411

[44] Stephan, A., Stadelmann, V.A., Leunig, M., & Impellizzeri, F. M. (2021). Measurement properties of promis short forms for pain and function in total hip arthroplasty patients.* Journal of Patient-Reported Outcomes, 5*. 10.1186/s41687-021-00313-1 10.1186/s41687-021-00313-1 PMC8165047 34056667

[45] Carle, A. C., Riley, W., Hays, R. D., & Cella, D. (2015). Confirmatory factor analysis of the patient reported outcomes measurement information system (promis) adult domain framework using item response theory scores.* Medical Care, 53*. 10.1097/MLR.0000000000000413 10.1097/MLR.0000000000000413 PMC4750392 26366521

[46] Wertli, M. M., Aegler, B., Mccabe, C. S., Grieve, S., Llewellyn, A., Schneider, S., Bachmann, L. M., & Brunner, F. (2023). Resilience in patients with complex regional pain syndrome 1-a cross-sectional analysis of patients participating in a cross-sectional cohort study.* Pain Medicine (United States), 24*. 10.1093/pm/pnad055 10.1093/pm/pnad055 PMC10472489 37154698

[47] Roser, K., Baenziger, J., Ilic, A., Mitter, V.R., Mader, L., Dyntar, D., Michel, G., & Sommer, G. (2023). Health-related quality of life before and during the covid-19 pandemic in Switzerland: a cross-sectional study.* Quality of Life Research, 32*. 10.1007/s11136-023-03414-0 10.1007/s11136-023-03414-0 PMC10119820 37084000

[48] Benz, T., Lehmann, S., Elfering, A., Sandor, P. S., & Angst, F. (2021). Comprehensiveness and validity of a multidimensional assessment in patients with chronic low back pain: a prospective cohort study.* BMC Musculoskeletal Disorders, 22*. 10.1186/s12891-021-04130-x 10.1186/s12891-021-04130-x PMC7981999 33743669

[49] Roser, K., Mader, L., Baenziger, J., Sommer, G., Kuehni, C. E., & Michel, G. (2019). Health-related quality of life in Switzerland: normative data for the sf36v2 questionnaire.* Quality of Life Research, 28*. 10.1007/s11136-019-02161-5 10.1007/s11136-019-02161-5 PMC6571102 30848444

[50] Angst, F., Benz, T., Lehmann, S., Aeschlimann, A., & Angst, J. (2018). Multidimensional minimal clinically important differences in knee osteoarthritis after comprehensive rehabilitation: A prospective evaluation from the bad zurzach osteoarthritis study.* RMD Open, 4*. 10.1136/rmdopen-2018-000685 10.1136/rmdopen-2018-000685 PMC6203096 30402264

[51] Henchoz, Y., Baggio, S., N’Goran, A. A., Studer, J., Deline, S., Mohler-Kuo, M., Daeppen, J. B., & Gmel, G. (2014). Health impact of sport and exercise in emerging adult men: a prospective study.* Quality of Life Research: An International Journal of Quality of Life Aspects of Treatment, Care and Rehabilitation, 23*. 10.1007/s11136-014-0665-0 10.1007/s11136-014-0665-0 24609388

[52] Angst, F., Goldhahn, J., Drerup, S., Kolling, C., Aeschlimann, A., Simmen, B. R., & Schwyzer, H. K. (2012). Responsiveness of five outcome measurement instruments in total elbow arthroplasty.* Arthritis Care and Research, 64*. 10.1002/acr.21744 10.1002/acr.21744 22674818

[53] Impellizzeri, F. M., Mannion, A. F., Leunig, M., Bizzini, M., & Naal, F. D. (2011). Comparison of the reliability, responsiveness, and construct validity of 4 different questionnaires for evaluating outcomes after total knee arthroplasty.* Journal of Arthroplasty, 26*. 10.1016/j.arth.2010.07.027 10.1016/j.arth.2010.07.027 21074964

[54] Gerber, M., Hartmann, T., Brand, S., Holsboer-Trachsler, E., & Pühse, U. (2010). The relationship between shift work, perceived stress, sleep and health in Swiss police officers.* Journal of Criminal Justice, 38*. 10.1016/j.jcrimjus. 2010.09.005

[55] Angst, F., Goldhahn, J., Drerup, S., Aeschlimann, A., Schwyzer, H. K., & Simmen, B. R. (2008). Responsiveness of six outcome assessment instruments in total shoulder arthroplasty.* Arthritis Care and Research, 59*. 10.1002/art.23318 10.1002/art.23318 18311752

[56] Angst, F., Verra, M. L., Lehmann, S., & Aeschlimann, A. (2008). Responsiveness of five condition-specific and generic outcome assessment instruments for chronic pain.* BMC Medical Research Methodology, 8*. 10.1186/1471-2288-8-26 10.1186/1471-2288-8-26 PMC2386498 18439285

[57] Puhan, M. A., Gaspoz, J. M., Bridevaux, P. O., Schindler, C., Ackermann-Liebrich, U., Rochat, T., & Gerbase, M. W. (2008). Comparing a disease-specific and a generic health-related quality of life instrument in subjects with asthma from the general population.* Health and Quality of Life Outcomes, 6*. 10.1186/1477-7525-6-15 10.1186/1477-7525-6-15 PMC2279107 18279510

[58] Rogenmoser, M., Klaghofer, R., Meyer, R. P., Kappeler, U., Burki, H., Hausner, P., Buddeberg, C., Buchi, S., & Stoll, T. (2003). Effects of hip arthroplasty followed by inpatient rehabilitation on physical function and quality of life.* Praxis, 92*. 10.1024/0369-8394.92.37.1515 14528725

[59] Keller, S. D., Ware, J. E., Bentler, P. M., Aaronson, N. K., Alonso, J., Apolone, G., Bjorner, J. B., Brazier, J., Bullinger, M., Kaasa, S., Leplège, A., Sullivan, M., & Gandek, B. (1998). Use of structural equation modeling to test the construct validity of the sf-36 health survey in ten countries: Results from the iqola project.* Journal of Clinical Epidemiology, 51*, 1179–1188. 10.1016/s0895-4356(98)00110-3 9817136

[60] Daeppen, J. B., Krieg, M. A., Burnand, B., & Yersin, B. (1998). Mos-sf-36 in evaluating health-related quality of life in alcohol-dependent patients.* American Journal of Drug and Alcohol Abuse, 24*. 10.3109/00952999809019617 10.3109/00952999809019617 9849778

[61] Perneger, T. V., Etter, J. F., & Rougemont, A. (1997). Prospective versus retrospective measurement of change in health status: A community based study in Geneva, Switzerland.* Journal of Epidemiology and Community Health, 51*. 10.1136/jech.51.3.320 10.1136/jech.51.3.320 PMC1060480 9229064

[62] Müller, R., Segerer, W., Ronca, E., Gemperli, A., Stirnimann, D., Scheel-Sailer, A., & Jensen, M. P. (2022). Inducing positive emotions to reduce chronic pain: a randomized controlled trial of positive psychology exercises.* Disability and Rehabilitation, 44*. 10.1080/09638288.2020.1850888 10.1080/09638288.2020.1850888 33264568

[63] Jenewein, J., Moergeli, H., Meyer-Heim, T., Muijres, P., Bopp-Kistler, I., Chochinov, H. M., & Peng-Keller, S. (2021). Feasibility, acceptability, and preliminary efficacy of dignity therapy in patients with early stage dementia and their family. a pilot randomized controlled trial.* Frontiers in Psychiatry, 12*. 10.3389/fpsyt.2021.795813 10.3389/fpsyt.2021.795813 PMC8740176 35002810

[64] Rauen, K., Vetter, S., Eisele, A., Biskup, E., Delsignore, A., Rufer, M., & Weidt, S. (2020). Internet cognitive behavioral therapy with or without face-to-face psychotherapy: A 12-weeks clinical trial of patients with depression.* Frontiers in Digital Health, 2*. 10.3389/fdgth.2020.00004 10.3389/fdgth.2020.00004 PMC8521970 34713017

[65] Mohler-Kuo, M., Schnyder, U., Dermota, P., Wei, W., & Milos, G. (2016). The prevalence, correlates, and help-seeking of eating disorders in Switzerland.* Psychological Medicine, 46*. 10.1017/S0033291716001136 10.1017/S0033291716001136 27444809

[66] Skevington, S. M., Lotfy, M., & O’Connell, K. A. (2004). The World Health Organization’s WHOQOL-BREF quality of life assessment: Psychometric properties and results of the international field trial a Report from the WHOQOL. *Group*. 10.1023/B:QURE.0000018486.91360.00 10.1023/B:QURE.0000018486.91360.00 15085902

[67] Husmann, A., Escorpizo, R., & Finger, M. E. (2020). Examining work-related functioning in a physical therapy outpatient clinic: Validity and reliability of the work rehabilitation questionnaire (worq).* Journal of Occupational Rehabilitation, 30*. 10.1007/s10926-019-09857-y 10.1007/s10926-019-09857-y 31468299

[68] Finger, M. E., Escorpizo, R., & Tennant, A. (2019). Measuring work-related functioning using the work rehabilitation questionnaire (worq).* International Journal of Environmental Research and Public Health, 16*. 10.3390/ijerph16152795 10.3390/ijerph16152795 PMC6696256 31387320

[69] Finger, M. E., Wicki-Roten, V., Leger, B., & Escorpizo, R. (2019). Cross-cultural adaptation of the work rehabilitation questionnaire (worq) to French: A valid and reliable instrument to assess work functioning. *Journal of Occupational Rehabilitation,**29*, 350–360. 10.1007/s10926-018-9795-5 29946812 10.1007/s10926-018-9795-5

[70] Ware, J. E., & Sherbourne, C. D. (1992). The mos 36-item short-form health survey (sf-36): I. conceptual framework and item selection.* Medical Care, 30*. 10.1097/00005650-199206000-00002 1593914

[71] Foundation, E.R.: EuroQol Research Foundation. EQ-5D-3L User Guide (2018). https://euroqol.org/publications/user-guides

[72] Cella, D., Riley, W., Stone, A., Rothrock, N., Reeve, B., Yount, S., Amtmann, D., Bode, R., Buysse, D., Choi, S., Cook, K., DeVellis, R., DeWalt, D., Fries, J. F., Gershon, R., Hahn, E. A., Lai, J.-S., Pilkonis, P., Revicki, D., Rose, M., Weinfurt, K., & Hays, R. (2010). Initial adult health item banks and first wave testing of the patient-reported outcomes measurement information system (promis ™) network: 2005–2008 on behalf of the promis cooperative group.* Journal of Clinical Epidemiology, 63*. 10.1016/j.jclinepi.2010.04.011 PMC2965562 20685078

[73] Perneger, T. V., Leplège, A., & Etter, J. -F. (1999). Cross-cultural adaptation of a psychometric instrument: Two methods compared.* Journal of Clinical Epidemiology 52*(11), 1037–1046 (1999). 10.1016/S0895-4356(99)00088-8 10526997 10.1016/s0895-4356(99)00088-8

[74] Spitale, G., Glässel, A., Tyebally-Fang, M., Dorey, C.M., Biller-Andorno, N. (2023). Patient narratives—a still undervalued resource for healthcare improvement.* Swiss Medical Weekly, 153*. 10.57187/smw.2023.40022 10.57187/smw.2023.40022 36787439

[75] Sprachen in der Schweiz (2022). https://www.bfs.admin.ch/bfs/de/home/statistiken/bevoelkerung/sprachen-religionen/sprachen.html

[76] McKown, S., Acquadro, C., Anfray, C., Arnold, B., Eremenco, S., Giroudet, C., Martin, M., & Weiss, D. (2020). Good practices for the translation, cultural adaptation, and linguistic validation of clinician-reported outcome, observer-reported outcome, and performance outcome measures.* Journal of Patient-Reported Outcomes*. 10.1186/s41687-020-00248-z 10.1186/s41687-020-00248-z PMC7642163 33146755

[77] Elsman, E. B. M., Baba, A., & Offringa, M. (2024). PRISMA-COSMIN 2024: New guidance aimed to enhance the reporting quality of systematic reviews of outcome measurement instruments. *International Journal of Nursing Studies,**160*, 104880. 10.1016/j.ijnurstu.2024.104880 39276710 10.1016/j.ijnurstu.2024.104880

