# Supplementary material for: A COSMIN systematic review of generic patient-reported outcome measures in Switzerland
Source: Qual Life Res. 2025 Apr 7;34(7):1869–84. doi: 10.1007/s11136-025-03942-x (PMC12182498; doi:10.1007/s11136-025-03942-x)
Supplement: Supplementary file 1 — Supplementary file1 (DOCX 82 kb) [file 11136_2025_3942_MOESM1_ESM.docx]

# Appendix A Search strategies

| **Table A1** |  | |
| --- | --- | --- |
| Oxford + Terwee Exclusion Filters*-* |  |  |
|  | PubMed | 1354 |
| (Swiss OR Switzerland) & “patient-reported outcome*” | Embase | 82 |
| “Swiss OR Switzerland” & “patient-reported outcome*”*-* | CINAHL | 62 |
| + All Generic PROM names (EXCLUDE children)*-* |  |  |
|  | PubMed | 409 (214) |
| “Swiss OR Switzerland” & “patient-reported outcome*” + All Generic PROMs (EXCLUDE children) | Embase | 41 (33) |
| “Swiss OR Switzerland” & “patient-reported outcome*” + All  Generic PROM names (EXCLUDE children)*-* | CINAHL | 10 (8) |
| + All Generic PROM names without “patient-reported outcome*” |  |  |
| (EXCLUDE children)*-* |  |  |
|  | PubMed | 5,386 |
|  |  | (2,361) |
| “Swiss OR Switzerland” + All Generic PROMs (EXCLUDE children) | Embase | 117 (65) |
| “Swiss OR Switzerland” & without “patient-reported outcome*” + | CINAHL | 522 (237) |
| All Generic PROM names (EXCLUDE children)*-* |  |  |
|  | Cochrane*-* | 132 |
|  | PsycInfo | 2,063 |
|  | Web of Sci*-* | 2,311 |

22

All Generic PROMs for keyword search (supported from the Excel file from [[18](#_bookmark28)]*-*):*-* (“Medical Outcomes Study Short Form” OR SF-36 OR SF-12 OR “European Quality of Life Questionnaire” OR Euro QOL* OR EQ-5D* OR “Sickness Impact Profile” OR “Patient-Reported Outcome Measurement Information System” OR PROMIS*OR “Patient-health questionnaire” OR PHQ-15* OR “Perceived Quality of Life Scale” OR PQoL* OR “Patient Global Impression of Severity Scale” OR PGI* OR “ICHOM Overall Adult Health” OR OAH* OR “Nottingham Health Profile” OR NHP* OR “Primary Care Outcomes Questionnaire” OR PCOQ OR “Long-term Conditions Questionnaire” OR LTCQ OR “Oxford Participation and Activities Questionnaire” OR Ox-PAQ* OR “The impact on life questionnaire” OR IoL OR “World Health Organization Disability Assessment Schedule*” OR WHODAS* OR “WHO (Five) Well-Being Index” OR WHO-5* OR “World Health Organization Quality of Life” OR WHOQOL*)

*-*

# Appendix B Descriptive data

**Fig. B1**: A bar chart of g*-*e*-*n*-*e*-*r*-*ic*-* PROM usage through time*-*. *-*T*-*h*-*e*-*s*-*e*--*P*-*R*-*O*--*M*--*w*--*e*-*r*-*e*-*t*-*h*-*e top 5 most frequently used gPROM in the selected sample (k = 299). Some studies implemented more than one gPROM.

10

PROMIS−29 & SF (k = 4)

EQ−5D−3L & EQ−5D−5D (k = 64)

SF−36 & SF−12 (k = 191)

5

0

10

5

0

| WHOQOL−BREF & SF (k = 37) | | | | | | | | | | | | |
| --- | --- | --- | --- | --- | --- | --- | --- | --- | --- | --- | --- | --- |
|  |  |  |  |  |  |  |  |  |  |  |  |  |

10

5

0

| PRISM (k = 5) | | | | | | | | | | | | |
| --- | --- | --- | --- | --- | --- | --- | --- | --- | --- | --- | --- | --- |
|  |  |  |  |  |  |  |  |  |  |  |  |  |

10

5

0

10

5

0

1995

2000

2005

2010

2015

2020

23

# Appendix C Measurement properties

**[Added version of the PROM as well as adjusted the paper in chronological order. ]**

**Table C2**: Measurement properties’ ratings from individual study for each PROM. Studies arranged from old to new order ^∗^ evidence available for the domain of pain and function in PROMIS-29. ^∗∗^ indicates evidence available for the physical a domains in WHOQOL-BREF.

Version Measurement property Methodological quality Rating

EQ-5D

P

| Clouth J et al. [[41](#_bookmark51)] | EQ-5D-3L | Structural validity | very good | + |
| --- | --- | --- | --- | --- |
| Perneger et al. [[40](#_bookmark50)] | EQ-5D-3L | Construct validity | doubtful | - |
| Staerkle and Villiger [[39](#_bookmark49)]  Fankhauser et al. [[37](#_bookmark47)] Mannion et al. [[36](#_bookmark46)]  Impellizzeri et al. [[38](#_bookmark48)] Luthy et al. [[35](#_bookmark45)] | EQ-5D-3L  EQ-5D-3L EQ-5D-3L  EQ-5D-3L EQ-5D-3L | Reliability  Measurement error Construct validity Responsiveness Construct validity Responsiveness Reliability Measurement error Construct validity Measurement error Responsiveness Construct validity | doubtful  doubtful doubtful doubtful adequate very good doubtful doubtful adequate doubtful very good doubtful | -  ?/-  +  +  +  +  +  ?/-  +  ?  +  + |
| Marti et al. [[34](#_bookmark44)]  Marks et al. [[33](#_bookmark43)]  Giesinger et al. [[32](#_bookmark42)] Wendelspiess et al. [[31](#_bookmark41)] | EQ-5D-5L  EQ-5D-5L  EQ-5D-5L | Internal consistency  Reliability Measurement error Construct validity Responsiveness Reliability Measurement error Construct validity Responsiveness Construct validity Responsiveness Responsiveness | doubtful  adequate adequate adequate very good inadequate adequate very good very good adequate adequate adequate | +  +  -  +  +  ?  -  +  +  +  +  + |
| Frei et al. [[30](#_bookmark40)] | EQ-5D-5L | Responsiveness | very good | - |
| ROMIS-29  Carle et al. [[46](#_bookmark56)] Structural validity very good + Internal consistency very good +  Reliability adequate +  Measurement error doubtful -  Construct validity very good +  Responsiveness very good - | | | | |

Stephan et al. [[45](#_bookmark55)] ∗

| Internal consistency | very good | + |
| --- | --- | --- |
| Reliability | very good | + |
| Measurement error | adequate | ? |
| Construct validity | adequate | + |
| Responsiveness  24 | adequate | + |

| Elsman et al. [[42](#_bookmark52)] | PROMIS-29 v2.1 | Structural validity | very good | + |
| --- | --- | --- | --- | --- |
| Wertli et al. [[47](#_bookmark57)] | PROMIS-29 | Construct validity | doubtful | + |

Stephan et al. [[43](#_bookmark53)] ∗

| Internal consistency | very good | + |
| --- | --- | --- |
| Reliability | very good | + |
| Measurement error | adequate | + |
| Construct validity | adequate | + |
| Responsiveness | adequate | ? |

SF-36

Perneger et al. [[28](#_bookmark38)] SF-36 Structural validity very good ?

Internal consistency very good +

Construct validity very good + Perneger et al. [[62](#_bookmark72)] SF-36 Reliability doubtful -

Responsiveness doubtful + Keller et al. [[60](#_bookmark70)] SF-36 Structural validity very good + Daeppen et al. [[61](#_bookmark71)] SF-36 Internal consistency very good +

Reliability adequate + Rogenmoser et al. [[59](#_bookmark69)] SF-36 Construct validity doubtful +

Responsiveness doubtful + Angst et al. [[57](#_bookmark67)] SF-36 Reliability doubtful +

Responsiveness adequate + Puhan et al. [[58](#_bookmark68)] SF-36 Internal consistency very good +

Construct validity very good + Angst et al. [[56](#_bookmark66)] SF-36 Reliability doubtful +

Measurement error inadequate ?

Responsiveness very good + Gerber et al. [[55](#_bookmark65)] SF-12 Internal consistency very good +

Construct validity very good ? Impellizzeri et al. [[54](#_bookmark64)] SF-12 Cross-cultural validity adequate +

Reliability very good +

Measurement error very good ?

Construct validity very good +

Responsiveness adequate + Angst et al. [[53](#_bookmark63)] SF-36 Reliability doubtful +

Measurement error adequate ?

Responsiveness adequate - Henchoz et al. [[52](#_bookmark62)] SF-12 Construct validity doubtful + Angst et al. [[51](#_bookmark61)] SF-36 Reliability doubtful -

Responsiveness adequate + Roser et al. [[50](#_bookmark60)] SF-36 Internal consistency very good + Benz et al. [[49](#_bookmark59)] SF-36 Construct validity adequate +

Responsiveness very good + Roser et al. [[48](#_bookmark58)] SF-36 Construct validity very good +

Responsiveness very good +

WHOQOL

Skevington et al. [[67](#_bookmark77)] BREF Structural validity very good - Mannion et al. [[36](#_bookmark46)] BREF Reliability doubtful +

Measurement error doubtful ?

Construct validity adequate + Mohler-Kuo et al. [[66](#_bookmark76)] ∗∗ Construct validity adequate +

Rauen et al. [[65](#_bookmark75)] BREF Responsiveness doubtful +

25

| Jenewein et al. [[64](#_bookmark74)] | BREF | Internal consistency | adequate | ? |
| --- | --- | --- | --- | --- |
|  |  | Construct validity | adequate | + |
|  |  | Responsiveness | adequate | - |
| Mu¨ller et al. [[63](#_bookmark73)] | 5 items | Internal consistency | adequate | + |
|  |  | Responsiveness | adequate | - |
| WORQ |  |  |  |  |
| Finger et al. [[70](#_bookmark80)] |  | Internal consistency | inadequate | + |
|  |  | Reliability | adequate | + |
|  |  | Construct validity | adequate | + |
| Finger et al. [[69](#_bookmark79)] |  | Structural validity | very good | + |
|  |  | Internal consistency | very good | + |
| Husmann et al. [[68](#_bookmark78)] |  | Internal consistency | very good | + |
|  |  | Reliability | doubtful | ? |
|  |  | Construct validity | adequate | + |
